# Silicon Conical Structures by Metal Assisted Chemical Etching

**DOI:** 10.3390/mi11040402

**Published:** 2020-04-11

**Authors:** Oscar Pérez-Díaz, Enrique Quiroga-González

**Affiliations:** Institute of Physics, Benemérita Universidad Autónoma de Puebla, Puebla 72570, Mexico; oscpedi@gmail.com

**Keywords:** silicon cones, metal assisted chemical etching, transversal pores

## Abstract

A simple and inexpensive method to obtain Si conical structures is proposed. The method consists of a sequence of steps that include photolithography and metal assisted chemical etching (MACE) to create porous regions that are dissolved in a post-etching process. The proposed process takes advantage of the lateral etching obtained when using catalyst particles smaller than 40 nm for MACE. The final shape of the base of the structures is mainly given by the shape of the lithography mask used for the process. Conical structures ranging from units to hundreds of microns can be produced by this method. The advantage of the method is its simplicity, allowing the production of the structures in a basic chemical lab.

## 1. Introduction

Different techniques have been developed in order to produce Si structures to be used in applications such optoelectronics [1], energy storage [2], or sensors [3]. Among these techniques it is possible to find reactive ion etching (RIE), inductively-coupled plasma (ICP)-RIE, or chemical assisted ion beam etching (CAIBE); however, all of them require special equipment, like vacuum chambers or plasma generators. On the other hand, electrochemical etching of Si has proved to be a good option because it provides high control for Si dissolution. Nevertheless, etching complete wafers is complex, since high current densities in the range of amperes are required, which derive in undesired heating that makes necessary the use of high-quality cooling appliances.

On the other hand, the metal assisted chemical etching (MACE) technique does not require any special equipment or facilities, and makes possible the fabrication of complex structures [4,5,6]. MACE is performed by immersing a piece of semiconductor (commonly Si, but also other semiconductors like Ge [7] or III-V semiconductors [8,9] can be used), previously coated with a catalyst (usually the metals Au, Pt, or Ag [10]), in an HF based solution containing an oxidant agent (commonly H_2_O_2_, Na_2_S_2_O_8_ or KMnO_4_ [11,12]). The metal catalyzes the release of electronic holes from the oxidant and, depending on the potential energy difference between metal and semiconductor, it promotes their injection to the semiconductor. In the most common etching case, Si is oxidized beneath or around the metallized sections, and this oxide is dissolved by HF.

Despite it is possible to obtain different structures using MACE, the most of the reports on this technique indicate that the etching occurs most probably in crystallographic directions (there are fast etching planes and etch-stopper planes) or vertically [13]. However, it is also known that when the catalyst particles have diameters smaller than 40 nm, they produce pores either vertically or horizontally [14,15]. Furthermore, when MACE has been performed using particles with a dispersion of sizes from 10 to 400 nm, thin pores (with diameters below 100 nm) and wide pores (with diameters of hundreds of nanometers) were obtained vertically, while just thin pores were obtained horizontally. By eliminating the most of the particles of sizes below 100 nm, mainly vertical porosification was obtained [16]. The vertical porosification of large particles is due to the much larger contact area below than at the sides of these particles, considering that they are spherical. For lightly-doped p-type Si wafers, for example, a larger contact area means a larger injection of electronic holes, which speeds the etching rate up. The contact area plays an important role in MACE [17]. When the particles are smaller, the probability is the same to etch either vertically or horizontally. With this equal probability, it is possible to think about a porous section growing upon the time in a direction with an angle close to 45° with respect to the vertical (the same amount of particles may move in the x than in the y direction, at similar velocity, producing that dy/dx = 1). To the knowledge of the authors, there are no reports to date taking advantage of this effect.

In the present work, a methodology to obtain Si conical structures by MACE is proposed. It consist of creating porous regions by MACE (previously defined by photolithography), which grow in angle with the time (due to lateral and vertical porosification obtained with catalyst particles smaller than 40 nm), and removing them afterwards. This methodology brings more flexibility to the MACE process. Additionally, it has the advantage of being simple, because the entire process could be performed in basic chemical labs without the need of complex facilities or equipment. Arrays of conical structures are important for different applications. They have been used as multielectrode sensing platforms for neuronal or cardiac tissue [18]. Additionally, arrays of complete or truncated cones have been used as antireflection layers [19] or to enhance the absorption of light [20], for different optical and optoelectronic applications such as solar cells. Moreover, such arrays have been used to modify the wetting properties of surfaces [21], achieving even super-repellency of hydrophobic surfaces [22].

## 2. Materials and Methods

**Figure 1:**
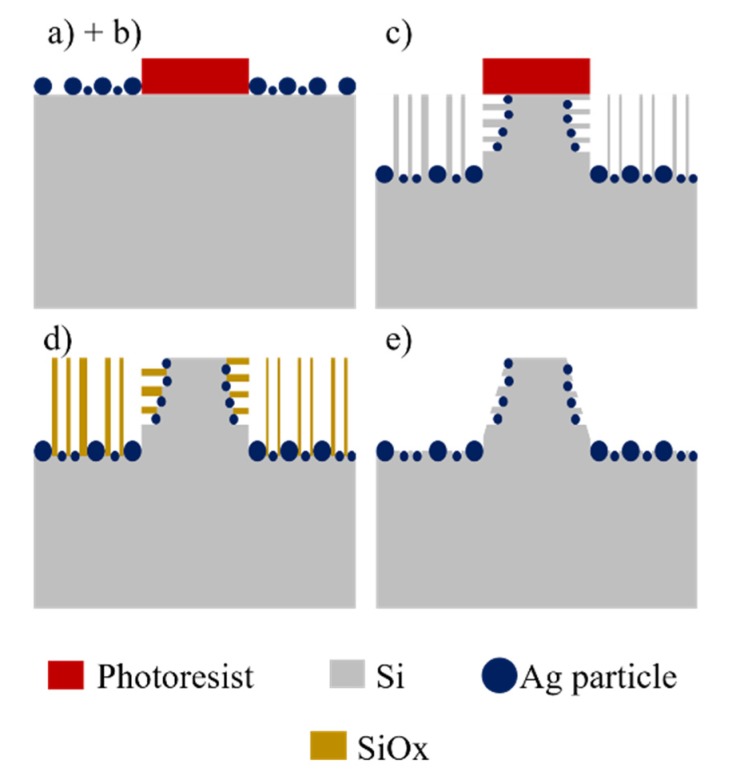
Schematic of the process to produce conical structures: (**a**) Photolithography, (**b**) deposition of Ag particles, (**c**) MACE, (**d**) dry oxidation, and (**e**) dissolution of oxide.

p-type (100) Si wafers with resistivity of 15–25 Ω·cm were used as starting material. The fabrication procedure to obtain the conical structures of the present work consists of a sequence of steps: (a) Photolithography, (b) chemical deposition of Ag particles, (c) MACE etching, (d) dry oxidation of the porosified sections, and (e) dissolution of oxide. Alternatively, at the end of the process one can also dissolve the Ag particles in solutions of HNO_3_. The steps are schematized in Figure 1.

A quadratic pattern of circles was transferred by photolithography to a film of photoresist previously deposited on the Si wafers. The photoresist acts as masking layer for the metal deposition. The metallization can be done as sophisticated and controlled as in the case of thin film deposition by sputtering [23,24,25], or as simple as in the case of chemical deposition of metal particles using just a beaker [26]. For this report, it was used the simplest case. Ag particles were chemically deposited on the uncovered sections of Si by immersion in a solution 0.1 mM of AgNO_3_ in a mixture of HF (48%), H_2_O_2_ (30%) and H_2_O, in a proportion 2:3.4:94.6 *v*/*v*. High-density polypropylene beakers were used for this and all the subsequent processes with HF, since that material endures adequately this acid. The deposition time was 90 s, being performed in an ultrasonic bath in order to obtain a homogeneous distribution of particles.

The etching process was performed using an aqueous solution containing HF (48%), H_2_O_2_ (30%) as oxidant agent [27], and deionized water (DI), in a proportion 4:7:40 *v*/*v* at 30 °C. The etching time was 5 h. With this process, porous Si sections were obtained. In order to obtain the final structures, the porous sections of the Si samples need to be dissolved. To accomplish this, it is possible to use anisotropic [28,29] or isotropic chemical etching techniques [30]; however, in order to dissolve mainly the porous sections without important modification of the shape of the remaining Si, those techniques were avoided in this work. The samples were submitted to thermal oxidation at 850 °C under O_2_ flux (1 sccm) for 3 h. With this process, the porous Si sections were oxidized. To dissolve the oxide, the samples were immersed in a solution of HF (48%) and H_2_O in a proportion 1:9 *v*/*v* for 60 s. Silicon oxides are highly soluble in HF based solutions [31]. The final structures were analyzed with a JEOL JSM-7500F (Tokyo, Japan) field emission scanning electron microscope.

## 3. Results and Discussion

**Figure 2:**
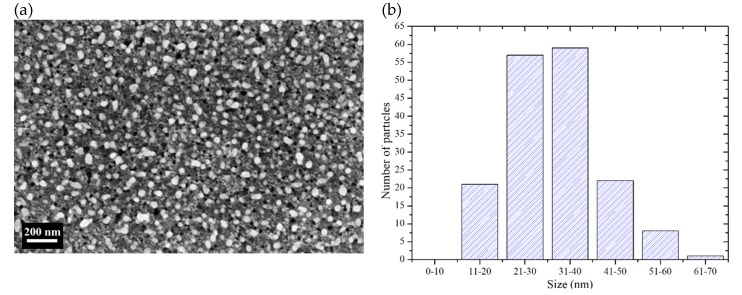
(**a**) SEM micrograph of the surface of a Si sample after deposition of Ag particles. (**b**) Size distribution of the Ag particles of the deposits.

Figure 2a shows a SEM (scanning electron microscope) micrograph of a Si sample after Ag deposition. The Ag particles are the white sections in the micrograph. Their shape is semi-spherical, but sometimes the particles coalesce giving rise to ovoidal forms. Semi-spherical shapes are commonly obtained when depositing using low concentrated AgNO_3_ solutions [32]; the particles nucleate and start to grow until they coalesce and could form dendrites at longer deposition times [16]. It is also important to note that the particles are encrusted in Si. This happens because of the use of H_2_O_2_ during the deposition process: The Ag particles deposit on Si and catalyze the etching of Si at the same time, in the presence of the oxidant. However, the trenches are shallow because the deposition time is short (90 s). The deposited Ag particles have diameters in the range of 10 to 70 nm. A histogram of the particle size distribution (measured from SEM micrographs of the deposits) is presented in Figure 2b. As can be seen, the most of the particles have sizes below 40 nm. It was intended to have particles with sizes below 40 nm taking into account previous studies that suggest that with particles of those sizes the probability of etching vertically or horizontally is similar [16,18,19].

**Figure 3:**
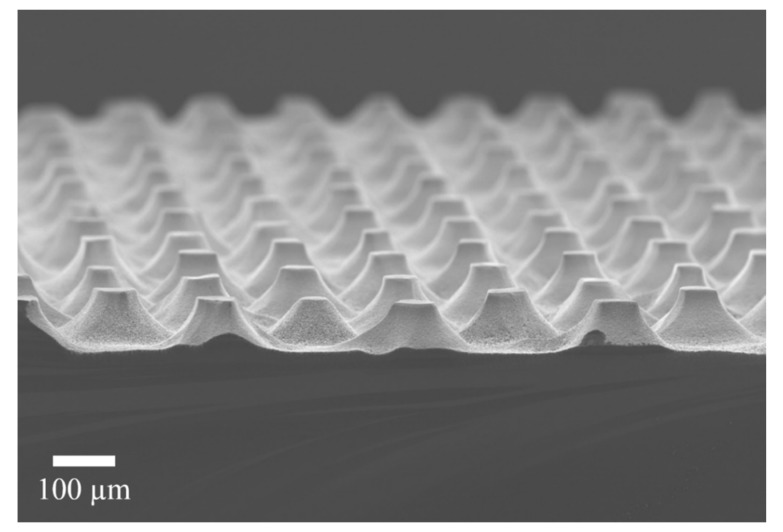
SEM micrograph of the obtained structures after the whole process. An array of truncated cones is evident.

Figure 3 shows a SEM micrograph of the structures obtained after the MACE process, dry oxidation and oxide dissolution. As can be observed, the structures are arrays of truncated cones. The bases and tops of the cones differ a bit from the circular shape. The diameters of the cones are 52 ± 5 µm for the top part and around 120 µm for the bottom part. They have a height of 60 µm.

**Figure 4:**
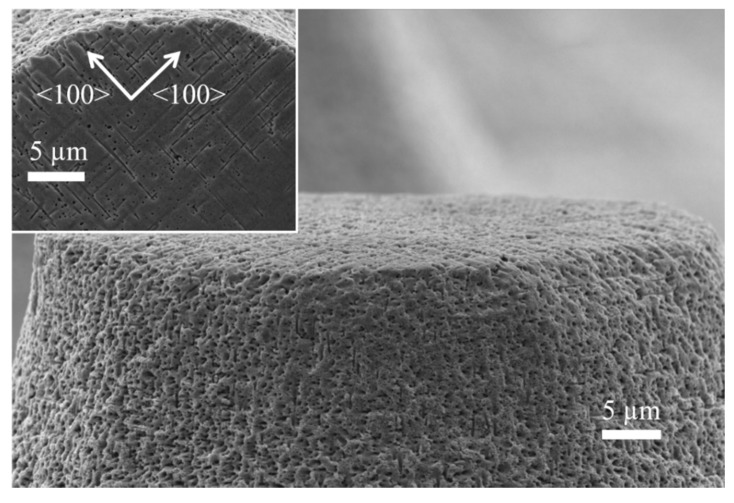
SEM micrograph closing up at the wall of the cones. Inset: Top view of the cones.

Figure 4 shows a close-up to the structures. The walls of the cones is rough, with apparent porosity. This is an indication that the oxidation time was not enough to oxidize the whole porous Si sections. Because of this, the porous sections could not be completely dissolved during the last treatment in HF solutions (that dissolve SiO_2_). However, the porosity of the cone walls is a good indication of the transversal porosification. Taking a look at the surface of the cones (inset of Figure 4) helps confirming the existence of transversal pores. They grow in the <100> directions. In principle, one would not expect pores exactly at the surface; however, Ag particles may grow through the photoresist (the photoresist is partially permeable to Ag^+^ ions during the deposition of Ag particles). It is important to mention that the photoresist used for the experiments of the present work is not HF resistant; nevertheless, it stands enough time for the Ag deposition, and it starts detaching during the etching process. It is not necessary that the photoresist stands the whole etching time, since no masking layer is necessary for this process (the etching rate in sections with Ag is hundreds of times faster than the etching rate in sections without catalyst). The few Ag particles grown beneath the photoresist could move in the X-Y plane during the etching process due to the availability of etchant in the surface (the photoresist does not stand HF, and the acid could diffuse through or below it); for this reason, it is possible to see transversal pores exactly at the surface.

**Figure 5:**
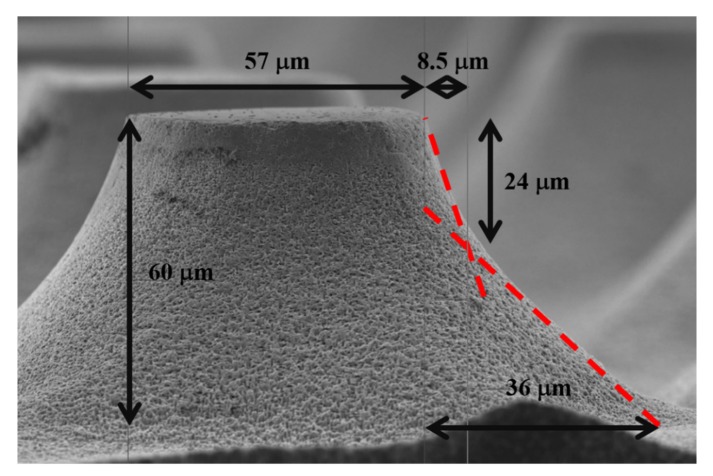
SEM micrograph of a truncated cone indicating its dimensions. The dashed lines indicate the two slopes of the cone walls.

Taking a closer look to one entire truncated cone (see Figure 5), one can observe two different slopes of the cone walls. Going up to down, the first slope is of 2.8, while the second is of 1.3. The steep first slope is given by an excess of etchant; thus, the etching process is reaction-rate limited. As could be observed in the histogram of Figure 2, there is a good number of particles larger than 40 nm. Those particles have a higher probability of etching vertically. As they offer larger areas to catalyze the decomposition of H_2_O_2_, they inject a larger number of electronic holes to the semiconductor enabling a faster etching rate than with the smaller particles. It is known that the one dimensional (vertical) etching rate increases with the catalyst particle size (in particular with the coverage area of the catalyst) [10], but until certain limit of sizes, when the mass transfer beneath the catalyst particles is limited, and the etching rate starts to decrease [11]. After the first 24 µm of etching in depth, the process is diffusion limited. It is common to observe diffusion limitation during a MACE process [33]. In this way, the etching process is mainly controlled by the availability of etchant, and the effect of the particle size is secondary. The difference between the vertical and the horizontal etching rate is about 30% in this depth range (27.5 µm of lateral etching vs. 36 µm of vertical etching, producing a slope of 1.3). The difference of etching rates in shallower depths is 180%. Following the tendency of the etching fronts, evoluting in angle, one can predict that if the MACE etching time is longer, complete conical structures (not truncated) could be obtained.

**Figure 6:**
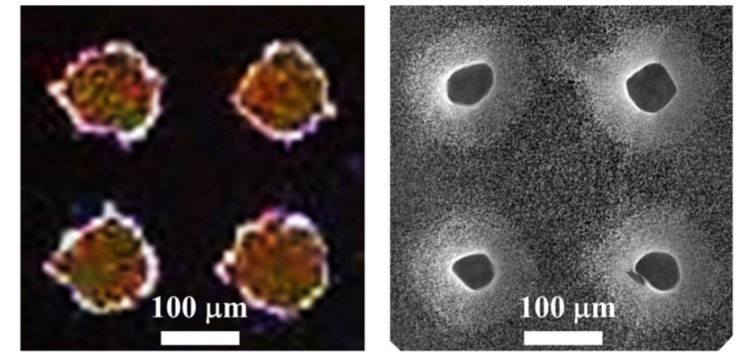
Left: Photograph of the pattern of photoresist used during the process. Right: SEM micrograph of a top view of the array of truncated cones.

Figure 6 shows a top view of the pattern of photoresist used during the etching process (photograph of the left), in contrast to the pattern of truncated cones obtained (SEM micrograph of the right). The photograph was captured with a portable optical microscope equipped with a CCD (charge-coupled device) camera. The dots of photoresist deviate a bit from the circular shape due to the resolution of the photomask, which was fabricated with a conventional paper printer. The diameter of the dots of the original pattern is of about 120 µm, with a pitch of 230 µm. The final structures have an upper diameter of 52 ± 5 µm, with a lower diameter in the range of 120 µm (as the original pattern).

**Figure 7:**
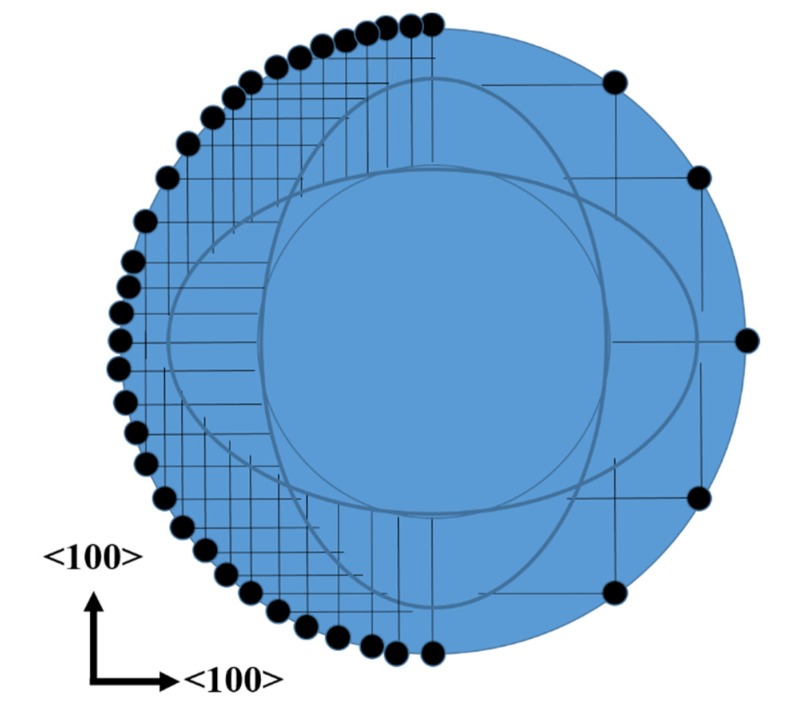
Schematic of the lateral pores observed from above. The black dots represent Ag particles, while the straight lines represent pores.

The fact that the top of the cones does not have the same shape than the dots of photoresist could be explained with the fact that the etching occurs mainly in <100> directions (see Figure 4). The etching profile of lateral pores, saw from above, is schematized in Figure 7. The dots of the figure represent Ag particles, while the straight lines represent the pores. All the straight lines have the same length, considering equal etching rates in the [100] and [010] directions. It is clear that the region without lines (pores) is not exactly a circle. It is also possible to observe that there are sections with lines (pores) just in one direction, thus the density of pores in those sections is just the half. Sections with pores in two directions can be oxidized faster, due to the higher density of pores, which provide larger surface areas to be oxidized. These oxidized sections can be easily removed in HF solutions. One could still observe pores in the walls of the cones of this work. This should indicate that the oxidation of all porous sections was not complete (the oxidation of the sections with pores in just one direction takes longer). If the oxidation time would be reduced even more, the cones would tend to have a flower-like shape (the sum of the sections with no lines and the sections with lines in one direction of Figure 7).

**Figure 8:**
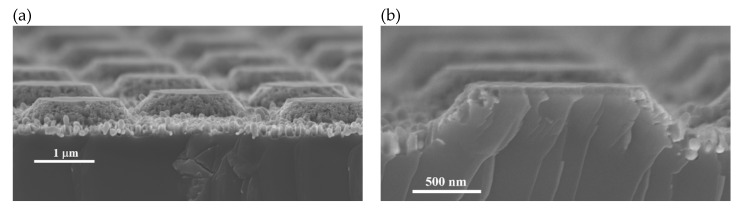
SEM micrographs of micron sized truncated cones produced by the methodology of this work. (**a**) Overview; (**b**) cross-section of a cone.

To prove that the proposed process to produce conical structures works also in the micron range, an experiment was performed using a mask with a quadratic array of circular dots of 1.5 µm, with pitch of 3 µm. A micrograph of the resulting structures is shown in Figure 8a. Figure 8b shows a micrograph of the cross-section of one of the micro-cones. As can be observed, no pores cross the structure, supporting the theory of cone formation. Despite the surface of the cones looks porous, the bulk is solid. The lines observed in the cross section are a cleavage artifact.

## 4. Conclusions

Transversal porosification of Si by MACE using Ag particles of sizes smaller of 40 nm has been used as basis to produce conical structures. At etching depths smaller than 24 µm, porosification is controlled by the reaction rate, producing steeper cone walls. Deeper etching is limited by the diffusion of the etchant, producing a reduction of the slope of the cone walls. Transversal porosification occurs mainly in the <100> direction. Due to this, the final cross-sectional shapes of the cones do not follow exactly the shape of the patterns of the photolithography mask. It was proved that the methodology works to produce conical structures of sizes from units to hundreds of micrometers, and it could be developed in basic chemical labs without complex equipment.
